# *In vivo* administration of dental epithelial stem cells at the apical end of the mouse incisor

**DOI:** 10.3389/fphys.2015.00112

**Published:** 2015-04-09

**Authors:** Giovanna Orsini, Lucia Jimenez-Rojo, Despoina Natsiou, Angelo Putignano, Thimios A. Mitsiadis

**Affiliations:** ^1^Orofacial Development and Regeneration, Centre for Dental Medicine, Institute of Oral Biology, University of ZürichZürich, Switzerland; ^2^Department of Clinical Sciences and Stomatology, Marche Polytechnic UniversityAncona, Italy

**Keywords:** mouse incisor, enamel, tooth, dental injury, dental pathology, regeneration, stem cells, cervical loop

## Abstract

Cell-based tissue regeneration is an attractive approach that complements traditional surgical techniques for replacement of injured and lost tissues. The continuously growing rodent incisor provides an excellent model system for investigating cellular and molecular mechanisms that underlie tooth renewal and regeneration. An active population of dental epithelial progenitor/stem cells located at the posterior part of the incisor, commonly called cervical loop area, ensures the continuous supply of cells that are responsible for the secretion of enamel matrix. To explore the potential of these epithelial cells in therapeutic approaches dealing with enamel defects, we have developed a new method for their *in vivo* administration in the posterior part of the incisor. Here, we provide the step-by-step protocol for the isolation of dental epithelial stem cells and their delivery at targeted areas of the jaw. This simple and yet powerful protocol, consisting in drilling a hole in the mandibular bone, in close proximity to the cervical loop area of the incisor, followed up by injection of stem cells, is feasible, reliable, and effective. This *in vivo* approach opens new horizons and possibilities for cellular therapies involving pathological and injured dental tissues.

## Introduction

The continuously erupting rodent incisor represents a suitable model system for studying cell proliferation, migration, differentiation, and mineral matrix deposition during development, homeostasis and regeneration of organs. Two of the hardest tissues of the body, the enamel and dentin, form as the outcome of interactions between oral epithelium cells and the cranial neural crest-derived mesenchyme during odontogenesis (Mitsiadis and Graf, 2009; Mitsiadis and Luder, 2011). The mineralized dental tissues are vulnerable to various external harmful agents, and to traumatic injuries that jeopardize tooth integrity. Loss of dental hard tissues in rodents caused by the frequent chewing and gnawing is balanced by constant cell divisions at the apical end of the incisor, allowing thus *de novo* enamel and dentin matrix formation by newly differentiated cells. Indeed, *in vivo* and *in vitro* cell tracing studies have shown that the cervical loops, which are located at the posterior part of the incisor, are niches for dental epithelial stem cells (DESCs) (Harada et al., 1999; Mitsiadis et al., 2007; Mitsiadis and Graf, 2009; Li et al., 2012). It has been demonstrated that DESCs are able to give rise to all epithelial cell layers of the incisor, including the enamel-forming layer of ameloblasts (Juuri et al., 2012; Biehs et al., 2013). Despite the obvious differences between rodent incisors and human teeth that include morphological, physiological and functional criteria there are fundamental similarities in dental hard tissue formation and structure in most of the species (Warshawsky et al., 1981; Jheon et al., 2013). However, damaged enamel cannot be repaired naturally in human teeth since ameloblasts are not present anymore after tooth eruption. Therefore, dental stem cells combined with tissue engineering products could be useful for the development of innovative strategies for cell-based dental tissue regeneration in the clinics (Mitsiadis et al., 2012).

To investigate the potential of DESCs in dental tissue regeneration and repair, we have applied an experimental model consisting of drilling a “window” in the alveolar bone of the mouse mandible, which overlies the apical part of the incisor. The creation of this bone window allows the injection of the DESCs at precise areas of the jaw, without affecting the overall physiology and masticatory attitudes of the animal. Here we demonstrate that this technique is successful and can be efficiently used to *in vivo* administer DESCs that could eventually be used for the repair of damaged or pathological dental tissues.

## Materials and methods

### Isolation of dental epithelial stems cells

- Dissect incisors from postnatal day 7 (PN7) ROSA26-EGFP mice. Incubate the incisors for 20 min at RT in Dispase (2 mg/ml) and DNAse (20 U/ml) solution in HBSS. Separate mechanically the epithelium from mesenchyme and dissect the cervical loop area.
- Add the tissues in 15 ml Falcon tubes with 14 ml of PBS/10% CS.
- Centrifuge at 300 g for 5 min.
- Remove supernatant.
- Add 1 ml of PBS.
- Centrifuge at 300 g for 5 min.
- Remove supernatant.
- Add 200 μl of 0.25% Trypsin (in PBS) and incubate 30 min at 37°C.
- Mix gently and pipet up and down vigorously.
- Add DNase I (2 U/ml) and incubate 5 min at 37°C.
- Add 700 μl of PBS/10% CS.
- Centrifuge at 300 g for 5 min.
- Remove supernatant.
- Add 1 ml of PBS.
- Centrifuge at 300 g for 5 min.
- Remove supernatant and resuspend DESCs in DMEM/F12 medium (1 ml).
- Filter the cells through 40 μm cell strainer.
- Count the cells.
- Pellet the cells at 300 g for 5 min.
- Resuspend DESCs in a solution of Growth Factor Reduced (GFR) Matrigel:PBS (1:8) at a concentration of 500000 cells/ml and keep them on ice.

### Animal surgery procedure

**Figure 1:**
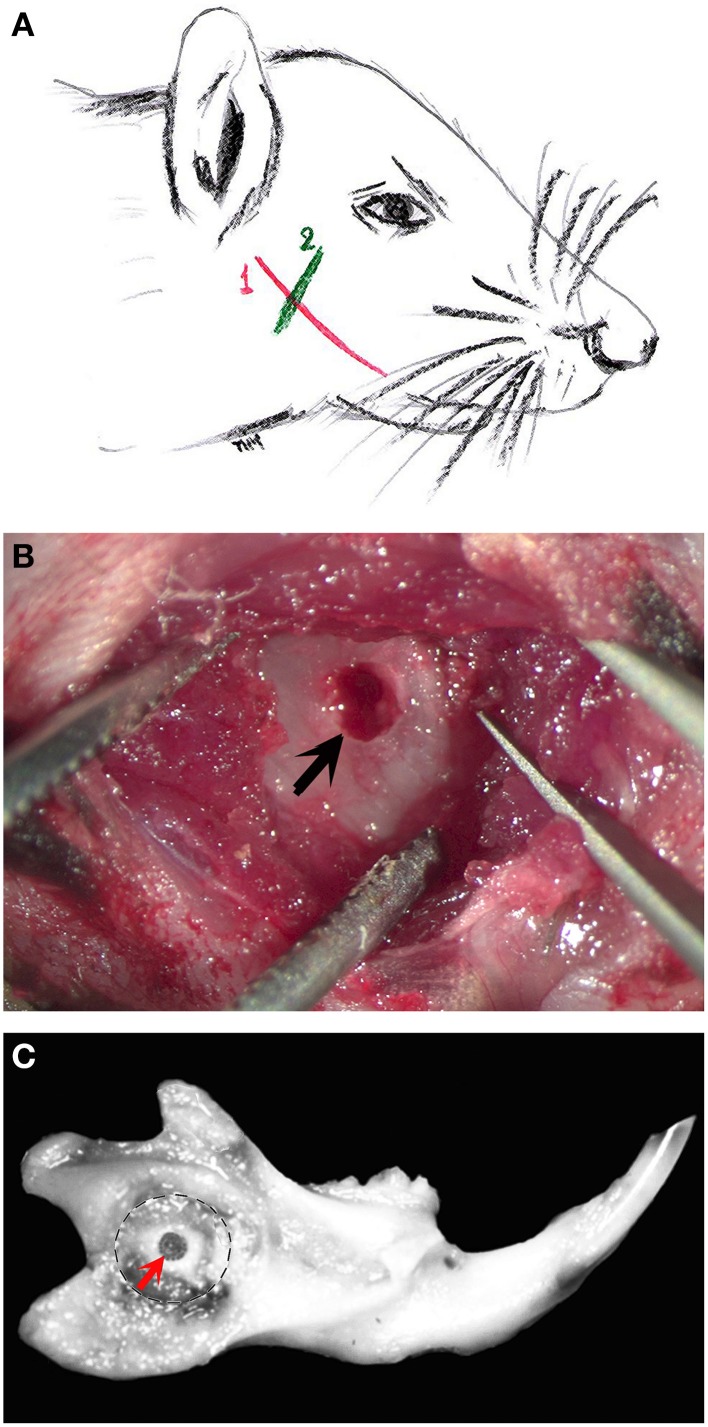
**The various steps of the bone “window” technique. (A)** Schematic representation of a mouse head showing the incision areas (in red and green colors) in order to expose the alveolar bone of the mouse mandible. The first incision line (in red color, 1) follows an imaginary line that joins the auditory meatus and the lip commissure. The incision is performed through the skin of the animal to expose the masseter muscle. The second incision line (in green color, 2) follows an imaginary line parallel to the posterior border of the mouse eye. This incision serves to separate the masseter muscle fibers in order to expose the alveolar bone in the proximity of the apical end of the incisor. **(B)** After incisions, the drilled bone “window” (arrow) is visible in the exposed mandibular alveolar bone. **(C)** Mouse dissected hemimandible, showing the drilled “window” approximately 2 mm from the incisure of the posterior mandibular border (red arrow).

- Use immunocompromised RAG1 -/- mice at 8–12 weeks of age.
- Before the surgery, inject intraperitoneally the anesthesia solution consisting of Ketamine (65 mg/kg body weight) and Xylacine (13 mg/kg body weight).
- Place the mice in the warming pad.
- Apply Vitamin A ointment (Bausch & Lomb) to the mice, in order to prevent eye dryness.
- Start the surgery when loss of response to reflex stimulation is observed.
- Make an incision about 4 mm long through the skin of the animal to expose the vestibular surface of the hemi-mandible, along an imaginary line joining the auditory meatus and the lip commissure, to access the muscle layer (Figure 1A).
- Separate the masseter fibers along their longitudinal axis using a scalpel blade, following an imaginary line parallel to the posterior border of the mouse eye (Figure 1A).
- Pay attention not to damage to the blood vessels and keep the muscle retracted using surgical tweezers.
- Use a periosteal separator to elevate the periosteum and expose the underlying bone surface.
- Drill the bone window approximately 2 mm from the posterior border of the ramus, estimating its position using a 1.8 mm dental Woodson condenser (Brassler, Montreal, QC, Canada).
- Use a slow-speed dental drill mounting a carbide round burr (Brassler) size 008 to make the bone window (Figure 1B).
- Irrigate using physiological saline solution during drilling.
- Use a Hamilton syringe Model 702N (with a 22-gauze needle) to inject 10 μl of the prepared solution of DESCs (5000 cells/injection).
- Seal the bone hole using dental canal sealer (AH Plus, Root Canal Sealing Material).
- Suture the masseter muscle using absorbable suture 6.0 (Ethicon Inc., Somerville, NJ).
- Suture the skin using non-absorbable silk suture 6.0 (Sherwood Davis & Geck, Wayne, NJ).
- Clean and disinfect the surgical site.
- Put mice onto a warming pad and observe until they reach consciousness.
- Follow pain management after surgery, by injecting Buprenorphine (0.1 mg/kg bodyweight) subcutaneously, every 6–8 h during the working day and orally administering it overnight, via the drinking water (buprenorphine 0.3 mg/ml are dissolved in 160 ml of water).
- Apply Buprenorphine treatment until day three after the surgery.

All mice were maintained and handled according to the Swiss Animal Welfare Law and in compliance with the regulations of the Cantonal Veterinary office, Zurich.

### Tissue processing

- At the desired time point of analysis, perfuse the mice with freshly prepared 4% Paraformaldehyde (PFA).
- Dissect the heads and postfix them in 4% PFA overnight at 4°C.
- Divide the heads in two equal halves along the longitudinal axis.
- Dissect the hemimandibles.
- Decalcify the samples during 6 ± 2 weeks using 10% EDTA at 4°C. Change the EDTA solution every 2–3 days.
- Process the samples for paraffin embedding.
- Section paraffin blocks at 5 μm and perform immunofluorescence against GFP antibody.
- Analyse the slides with Leica DM6000 FS microscope and take pictures with the Leica DFC350FX camera for the fluorescence imaging and the Leica DFC420C camera for bright-field imaging.

## Results

We have used immunocompromised (RAG1-/-) mice as recipients of DESCs in order to prevent the rejection of the transplanted cells. Thus, all mice recovered well and no complications were observed during the healing period.

**Figure 2:**
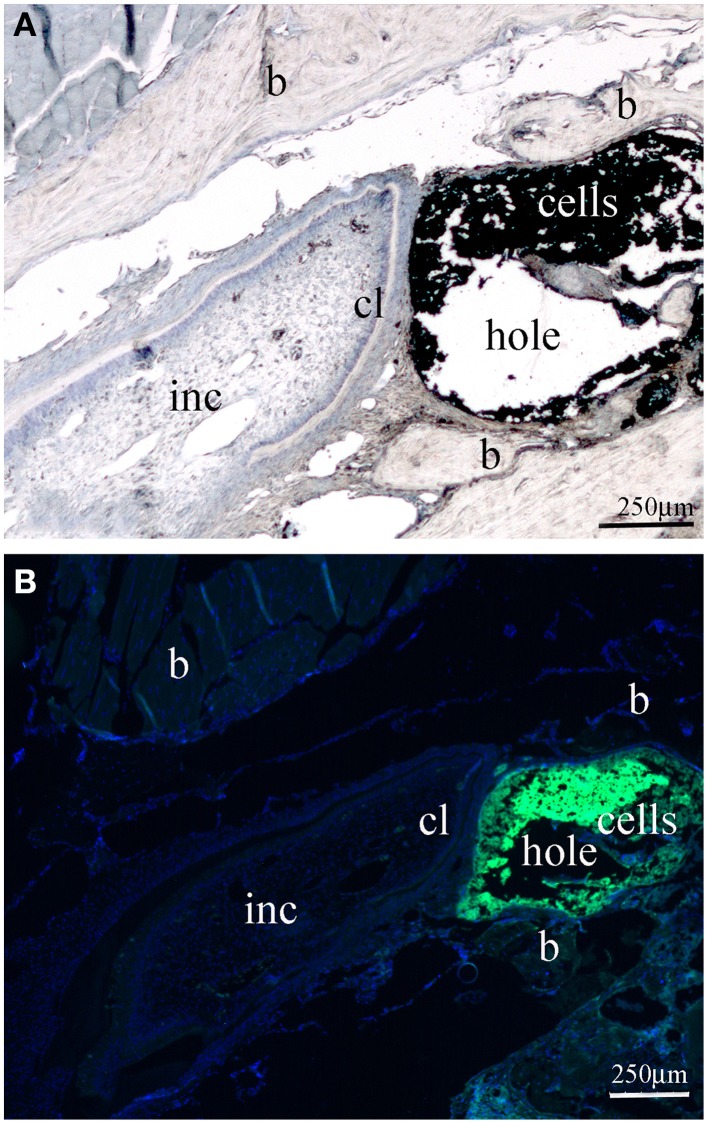
**Longitudinal sections through the mouse mandible after the creation of the bone “window” and the injection of GFP-positive dental epithelial stem cells. (A)** Light micrograph showing the area of the alveolar bone “window” in close proximity to the posterior end of the mouse incisor. **(B)** Fluorescence micrograph showing the GFP positive dental epithelial stem cells in the drilled hole of the alveolar bone. Abbreviations: b, bone; cl, cervical loop area; inc, incisor. Bars: 250 μm.

The appropriate position of the bone window was confirmed at time of hemimandibles dissection (Figure 1C). In all cases the bone windows performed at the labial mandibular bone were drilled very close to the apical part of the incisor. Histologically, there was no alteration of the enamel organ: the drilling did not disrupt the dental tissues and more precisely the external epithelial layer of the incisor (Figure 2A). Green fluorescence protein (GFP) positive DESCs were observed in the hole (Figure 2B), showing that the GFP-expressing DESCs were successfully delivered to the vicinity of the apical part of the mouse incisor.

## Discussion

Cell-based regenerative therapies consist of the *in vivo* administration of stem cells to patients (Mitsiadis et al., 2012). Stem cell transplantation has already been shown to be successful for the treatment of several damaged or pathological tissues. For instance, cultured human keratinocyte stem cells have been largely used for the treatment of patients with third-degree burns (Pellegrini et al., 2009). Similarly, human corneal regeneration has been achieved after transplantation of diverse sources of cells such as limbal stem cells (Rama et al., 2010) and oral mucosal epithelial cells (Burillon et al., 2012). Therefore, specific stem cell populations derived from different organs and tissues are extremely interesting for clinical tissue engineering applications. The present step-by-step protocol provides a comprehensive view of a novel experimental procedure for the isolation and local delivery of DESCs in precise areas of the mouse mandible. Isolation of DESCs was based on previous protocols for dental (Chavez et al., 2013, 2014) or other non-dental tissues (Smalley, 2010; De Marval et al., 2014).

Several earlier reports have demonstrated that the formation of a bone window in the rat mandible, where osmotic minipumps can be adapted, constitutes an efficient method for the local and continuous delivery of various substances (Orsini et al., 2001; Nanci et al., 2004), as well as for gene transfer purposes (Wazen et al., 2006). Here we have adapted these techniques in order to develop a new method for *in vivo* stem cells delivery into precise areas of the mouse incisor such as its apical part. This newly described approach would be useful to trace the *in vivo* fate of the DESCs after their injection, and further analyse their integration capacity within the dental tissues.

The bone window technique allows the administration of a relatively high number of stem cells *in situ* that will be necessary for tissue repair and regeneration. However, some caveats cannot be exclused when realizing this technique. For example, because of the confined and narrow space separating the alveolar bone and the underlying dental epithelium, inappropriate position of the hole can either damage the apical end of the incisor or perforate the thin alveolar bone. Another parameter that has to be taken into consideration is time. It is necessary to obtain an efficient strategy for controlling the time period that will eventually vary according to the quantity of injected DESCs. Future developments of this technique are the tracing of injected GFP-positive DESCs and their fate when will incorporate the dental tissues.

To date, this method can be considered a useful *in vivo* approach for delivering DESCs in the mouse incisor. This could lead to greater biologic responsiveness, since the administered cells can endogenously synthesize proteins that may continue to exert its effect *in situ*. Thus, this technique could be easily adapted for the needs of the practitioners in the future. For instance, a potential applications of this technique in humans could be the repair of alveolar bone defects or bone loss during periodontal disease. However, it is still a great challenge to find appropriate sources of cells that ideally could be *in vitro* expanded without losing their regenerative capacity and, in addition, do not cause rejection by the recipient's immune system once transplantated into the target tissue.

### Conflict of interest statement

The authors declare that the research was conducted in the absence of any commercial or financial relationships that could be construed as a potential conflict of interest.
